# *Enterovirus* 74 Infection in Children

**DOI:** 10.1371/journal.pone.0076492

**Published:** 2013-10-02

**Authors:** Matthew Peacey, Richard J. Hall, Jing Wang, Angela K. Todd, Seiha Yen, Jasmine Chan-Hyams, Christy J. Rand, Jo-Ann Stanton, Q. Sue Huang

**Affiliations:** 1 Clinical Virology, The Institute of Environmental Science and Research, National Centre for Biosecurity and Infectious Disease, Wellington, New Zealand; 2 Department of Anatomy, University of Otago, Dunedin, New Zealand; University of Malaya, Malaysia

## Abstract

Enterovirus 74 (EV74) is a rarely detected viral infection of children. In 2010, EV74 was identified in New Zealand in a 2 year old child with acute flaccid paralysis (AFP) through routine polio AFP surveillance. A further three cases of EV74 were identified in children within six months. These cases are the first report of EV74 in New Zealand. In this study we describe the near complete genome sequence of four EV74 isolates from New Zealand, which shows only limited sequence identity in the non-structural proteins when compared to the other two known EV74 sequences. As is typical of enteroviruses multiple recombination events were evident, particularly in the P2 region and P3 regions. This is the first complete EV74 genome sequenced from a patient with acute flaccid paralysis.

## Introduction

The genus Enterovirus (EV), family *Picornaviridae*, comprises more than 110 serotypes [1]. Most are known human pathogens and are classified as four species Enterovirus (EV)-A, EV-B, EV-C (includes poliovirus) and EV-D determined by phylogenetic analysis and differing from each other by >40% nucleotide divergence. The majority of *Enterovirus* infections are asymptomatic, but can cause a broad spectrum of disease such as acute respiratory illness, gastrointestinal illness and more serious manifestations, including aseptic meningitis, encephalitis and acute flaccid paralysis (AFP) [2].

The enterovirus genome comprises a single open reading frame translated into a polypeptide cleaved by viral proteases. The translated product has three domains; P1, P2, P3. The viral capsid proteins (VP4-VP1) are encoded in the P1 domain and provide the basis for classification of enteroviruses. Non-structural proteins 2A-C and 3A-D are respectively encoded in the P2 and P3 domains [3].

Recombination has been widely reported between polio strains and non-polio enteroviruses. Enterovirus species B has been shown undergo much more frequent recombination events than found for species A [1,4-11]. Recent studies have suggested recombination frequently occurs between independently evolving genomes creating new virus variants where the boundaries between lineages are not fixed [10]. Recombination is thought to occur through two mechanisms; 1. ‘copy choice’ where the viral polymerase switches template RNA molecules during minus-strand RNA synthesis and 2. a proposed non-replicative mechanism involving relatively unspecific covalent joining of RNA fragments [11].

*Enterovirus* 74 (EV74) is a rare viral pathogen usually infecting children, and has been known to cause AFP. EV74 was first proposed as a new serotype in the EV-B species by Oberste et al. in 2004 after sequencing the VP1 gene. The full genome of a 1975 Californian strain USA/CA75-10213 (AY556057) isolated from stool was sequenced providing a prototype for EV74 [12].

To date cases have been reported worldwide from the USA, Finland, India, China, France, Bangladesh, Iraq, Korea and the Central African Republic [12-16]. All patients were children ranging in age from 12 months to 14 years old and the manifestations of the different isolates varied. Of the thirteen cases where any patient data was available, four patients suffered acute flaccid paralysis (AFP), two patients presented with fever and two had acute gastroenteritis. Pneumopathy, seizures and headaches were reported separately in single cases, and symptoms in the remainder of cases are not reported [12,17-19].

EV74 was first identified in New Zealand in 2010, in a 2 year old child with AFP during routine polio AFP surveillance by partial sequencing of the VP1 gene. A further three cases of EV74 were identified in children within a six month period, but none were known to have resulted in AFP. Deep sequencing was employed to determine the full genome of the New Zealand EV74 isolate, resulting in AFP, revealing significant recombination. Full genome sequences for the additional three EV74 isolates from New Zealand were then obtained and compared revealing multiple recombination events.

## Methods

### Patients and specimen collection

Samples were collected by the National Poliovirus and Enterovirus Reference Laboratory at the Institute of Environmental Science and Research (ESR), approved by the Ministry of Health, for public health purposes. Data was analysed anonymously. Clinical specimens or cell culture isolates from un-typed enteroviruses occurring in four major hospitals (based in Auckland, Waikato, Wellington and Christchurch) are received year round as part of the New Zealand enterovirus surveillance network.

### Viruses and cells

Human rhabdomyosarcoma (RD) cells (Centers for Disease Control and Prevention, USA, passage 242-256) were propagated in 10% Hanks Minimal Essential Medium (Life Technologies) supplemented with 10% (v/v) fetal bovine serum (HyClone, New Zealand), 7.5% (v/v) sodium bicarbonate, 1% (v/v) 1M HEPES and Gentamicin (53 µg/ml, Pfizer), Streptomycin (13 U/ml, Life Technologies) and Penicillin (13 µg/ml, Life Technologies). Although EV74 isolates grew best in RD and MRC5 cells, good growth was also observed in L20B cells and limited growth in MEK cells.

### Faecal specimens

Chloroform extraction of faecal specimens was performed by combining a 0.5-0.8g of faecal material with 8 glass beads, and 400 µl of chloroform. The suspension was then shaken vigorously for 20 minutes prior to centrifugation at 2800 x *g* for 20 minutes. The supernatant was removed, 400 µl of chloroform added and before centrifugation at 2800 x *g* for 10 minutes. Again the supernatant was removed and centrifuged at 13000 x *g* for 5 minutes before re-suspension in phosphate buffered saline. The final suspension was inoculated into RD cell lines.

### Roche 454 enterovirus genome sequencing

Full-length genomic sequence of EV74 from strain NZ2284 was obtained by sequencing using a Roche GS FLX instrument. RNA from passage 1 viral culture was extracted using the MagMax Viral RNA kit (Life Technologies, Cat# AM1939) as per the manufacturer’s instructions, except without the inclusion of carrier RNA, and a final elution volume of 50µL. RNA concentration was determined to be too low (

< 1µg) for direct inclusion in the Roche GS FLX sequencer library preparation, so Multiple strand Displacement Amplification (MDA) using the Quantitect Whole Transcriptome Kit (Qiagen, Cat# 207043) was employed in combination with a SuperScript III First-Stand Synthesis kit, random hexamers and protocol(Life Technologies, Cat# 18080-051). Two amplification methods were trialled, differing only with the omission of the ligation step in one of the amplification reactions (both sets of data were used in the subsequent analysis). 500 ng of amplified DNA was nebulized and used to make a barcoded, GS FLX Rapid Library (Roche, Mannheim, Germany) according to the manufacturer’s instructions. Completed library length was between 500 bp and 900 bp with a concentration of 3.45 x 10^9^ molecules/µL. An emPCR reaction was performed using a library concentration of 2 cpb per oil giving approximately 500,000 enriched beads. These were loaded onto a GS Junior Titanium PTP plate and sequenced on a GS Junior instrument using Titanium chemistry (Roche). The utilities sffinfo21 and FASTX-Toolkit22 were used to remove adaptor and primer sequences and filter sequence based upon data quality. Subsequent BLAST search performance was enhanced because redundant sequence information was not submitted. Enterovirus short reads identified in a BLASTN search as EV74 sequence were assembled into 11 contigs using Roche Newbler de novo assembler (Roche) [20]. Further assembly and annotation was achiev

ed using Geneious 5.5.6 (Biomatters Limited, New Zealand).

### Sanger full-genome sequencing

All four New Zealand strains were sequenced as described previously (Lukashev, 2005). Nucleic acid was reverse transcribed into cDNA using random hexamers and the Superscript III First-Strand Synthesis System for RT-PCR. cDNA was then amplified using Platinum® PCR SuperMix (Life Technologies, Cat# 12532016). After an initial activation for 2 minutes at 94°C, a three-step cycling procedure was used consisting of; 40 cycles of denaturation at 94°C for 1 minute, annealing at 45°C for 1 minute and extension at 72°C for 2 minutes. A final extension took place at 72°C for 5 minutes. Samples were run on an E-Gel EX 2% precast agarose gel (Life Technologies, Cat# G501808). DNA bands of interest were extracted and purified using the QIAquick PCR Purification Kit (Qiagen, Cat# 28104) and sequenced using an ABI 3130XL automated DNA sequencer and ABI Big Dye v3.1 technology (Life Technologies).

### Phylogenetic analysis

New Zealand isolate sequences were deposited in the NCBI Genbank database and assigned accession numbers KC568446 (NZ45), KC568447, (NZ234), KC568448 (NZ2205) and KC568449 (NZ2284). These sequences were aligned with complete prototype EV-B genome sequences using Geneious 5.6.5 and CLUSTALW (gap open cost 15 and gap extend cost 6.66). See Table 1 for a complete list of EV-B viruses and accession numbers of New Zealand isolates used in this study. Phylogenetic trees were built with Geneious 5.6.5 using the neighbour-joining method and Jukes-Cantor evolution model. A bootstrap resampling method was used with 1,000 replicates. In-tree comments were added using Visio 2010 (Microsoft).

**Table 1:** Prototype Enterovirus B strains and New Zealand EV74 isolates.

**Type**	**Accession**	**Strain**			**Type**	**Accession**	**Strain**
CAV9	D00627	Griggs			E30	AF162711	Bastianni
CBV1	M16560	Not Found in NCBI			E31	AY302554	Caldwell
CBV2	AF081485	Ohio			E32	AY302555	PR-10
CBV3	AY896762	CBV3-11059-99			E33	AY302556	Toluca-3
CBV4	X05690	J.V.B. Benschoten			EV69	AY302560	Toluca-1
CBV5	AF114383	Faulkner			EV73	AF241359	CA55-1988
CBV6	AF105342	Schmit			EV74	AY556057	USA/CA75-10213
E1	AF029859	Farouk / ATCC VR-1038			EV74	JQ397329	Rikaze-136/XZ/CHN/2010
E2	AY302545	Cornelis			EV74	KC568446	NZ45
E3	AY302553	Morrisey			EV74	KC568447	NZ234
E4	AY302557	Pesacek			EV74	KC568448	NZ2205
E5	AF083069	Noyce			EV74	KC568449	NZ2284
E6	AY302558	D’Amori			EV75	AY556070	USA/OK85-10362
E7	AY302559	Wallace			EV77	AY843302	USA/TX97-10394
E9	X92886	Barty			EV79	AY843297	USA/CA79-10384
E11	X80059	Not Found in NCBI			EV80	AY843298	USA/CA67-10387
E12	X79047	prototype Travis			EV81	AY843299	USA/CA68-10389
E13	AY302539	Del Carmen			EV82	AY843300	USA/CA64-10390
E14	AY302540	Tow			EV83	AY843301	USA/CA76-10392
E15	AY302541	CH 96-51			EV84	DQ902712	CIV2003-10603
E16	AY302542	Harrington			EV85	JX898909	HTYT-ARL-AFP02F/XJ/CHN/2011
E17	AY302543	CHHE-29			EV86	AY843304	BAN00-10354
E18	HM777023	Kor05-ECV18-054cn			EV87	AY843305	BAN01-10396
E19	AY302544	Burke			EV88	AY843306	BAN01-10398
E20	AY302546	JV-1			EV97	AY843307	BAN99-10355
E21	AY302547	Farina			EV98	NC_013114	T92-1499
E24	AY302548	DeCamp			E100	NC_009887	BAN2000-10500
E25	AY302549	JV-4			EV101	AY843308	CIV03-10361
E26	AY302550	Coronel			EV107	NC_013115	TN94-0349
E27	AY302551	Bacon			PV	NC002058	Poliovirus 1 Mahoney
E29	AY302552	JV-10					

### Recombination Analysis

Aligned sequences were analysed using SimPlot version 3.5.1 [21] to identify areas of recombination. Similarity plots were created with a window size of 500 nt, step size of 20 nt, and the Jukes-Cantor model. Bootscan search settings were as follows; neighbour-joining method, Kimura evolution model and 100 replicates, with a window size of 500 nt and step size of 20 nt.

## Results

Four samples from EV74 infected patients were received within a six month period coinciding with the summer in New Zealand. All four cases came from the central North Island, within 400 km (250 miles) of each other (Table 2). Consistent with international reports of EV74, the patients were all children, ranging in age from 1 month to 10 years of age [12,17-19]. Three of the four patients were male and all patients presented to hospital with fever. Other symptoms included headache, diarrhoea, photophobia, rash and in one case AFP.

**Table 2:** Summary of data collected for EV74 infected patients.

**Strain**	**Date received**	**Gender**	**Age**	**Symptoms**	**Location**
NZ2205	4/11/2010	M	10 y	Headache, fever, photophobia, flu-like symptoms	Bay of Plenty
NZ2284	30/11/2010	M	2 y	AFP, fever, unable to stand	Bay of Plenty
NZ45	25/01/2011	F	1 m	Febrile illness	Gisborne
NZ234	28/04/2011	M	9 m	Diarrhoea, fever, rash	Hamilton

Deep sequencing of isolate NZ2284 yielded 60,274 sequences ranging between 53 bps and 1200 bps in length before quality trimming. A BLASTN and BLASTX search were performed on the quality-checked dataset which identified 43,824 (73%) of the 60,284 reads as EV and 5,435 reads (9%) specifically as EV74. Closer examination of EV74 BLASTN alignments revealed 39% of sequences aligned with the VP1 region. *De novo* assembly of all enterovirus sequence reads provided coverage of 91% of the EV74 genome with an average depth of 65.

A sanger–sequencing approach consisting of eight overlapping amplicons was employed to provide full genome sequences for all four New Zealand EV74 samples. All but 30-58 nt of the 5’ NTR was not determined for each EV74 strain with each sequence ranging in length from 7311-7359nt. Complete concordance was observed between deep-sequencing and sanger-sequencing data for isolate NZ2284.

**Figure 1:**
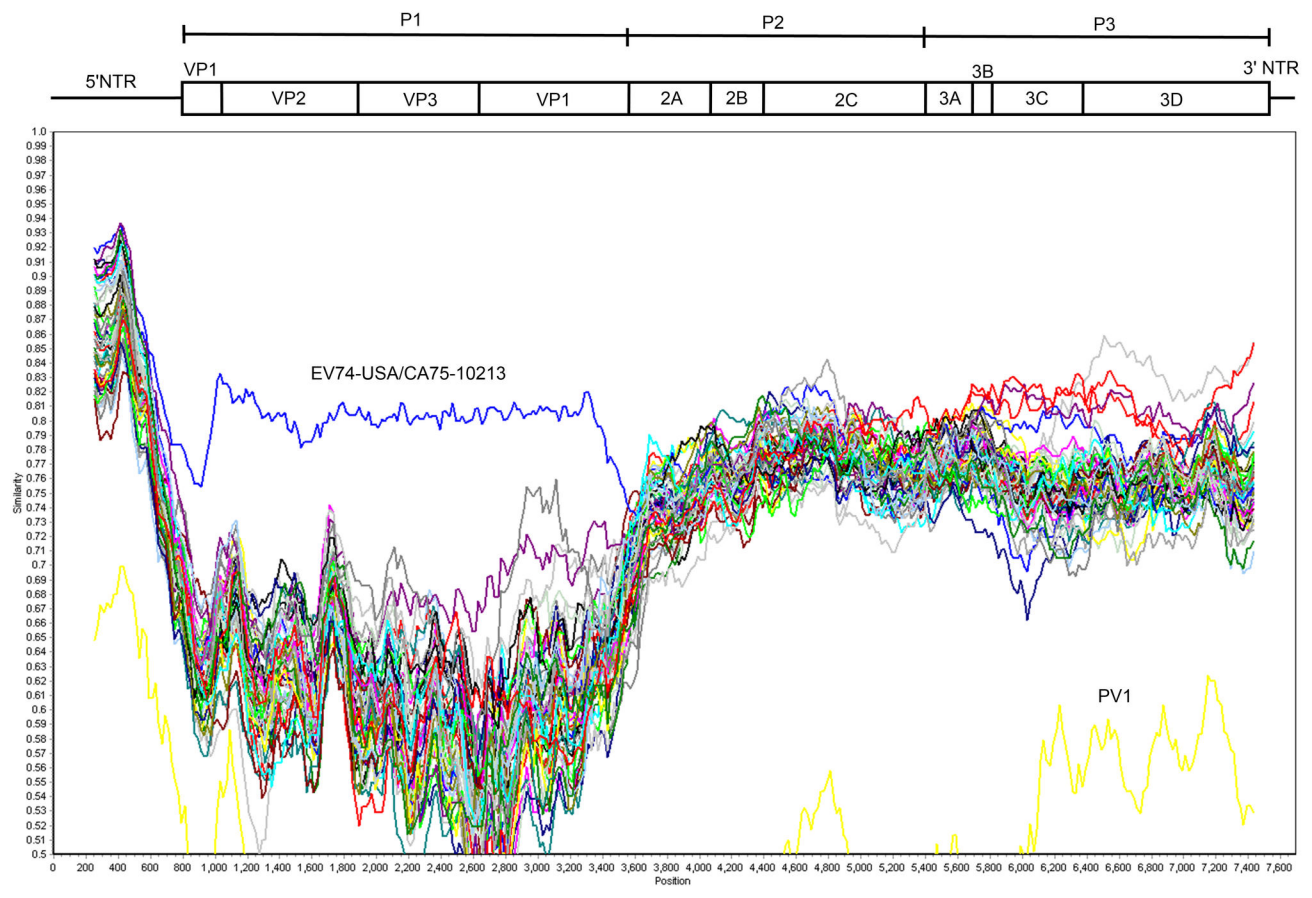
Similarity plot comparing NZ2284 with all available prototype EV B strains and PV1. Schematic above the graph indicates the position of each gene within the EV genome.

Similarity Plot, Bootscan analysis, pairwise sequence comparison and multiple alignments were used to examine the relationship of New Zealand isolates to the two existing full-length EV74 reference sequences available in the public databases. The four New Zealand EV74 isolates showed an average of 92.1% and 92.8% nucleotide sequence identity to the VP1 gene of EV74 reference genomes USA/CA75-10213 (AY556057) and Rikaze-136/XZ/CHN/2010 (JQ397329), respectively, confirming that EV74 was the correct genotype designation for these isolates (Figure 1). Whole genome comparison of all four sequenced New Zealand EV74 isolates showed an average nucleotide sequence identity of 98.7% to each other, indicating a common strain, but only 79.8% identity to USA/CA75-10213, and 80.9% identity to Rikaze-136/XZ/CHN/2010.

**Figure 2:**
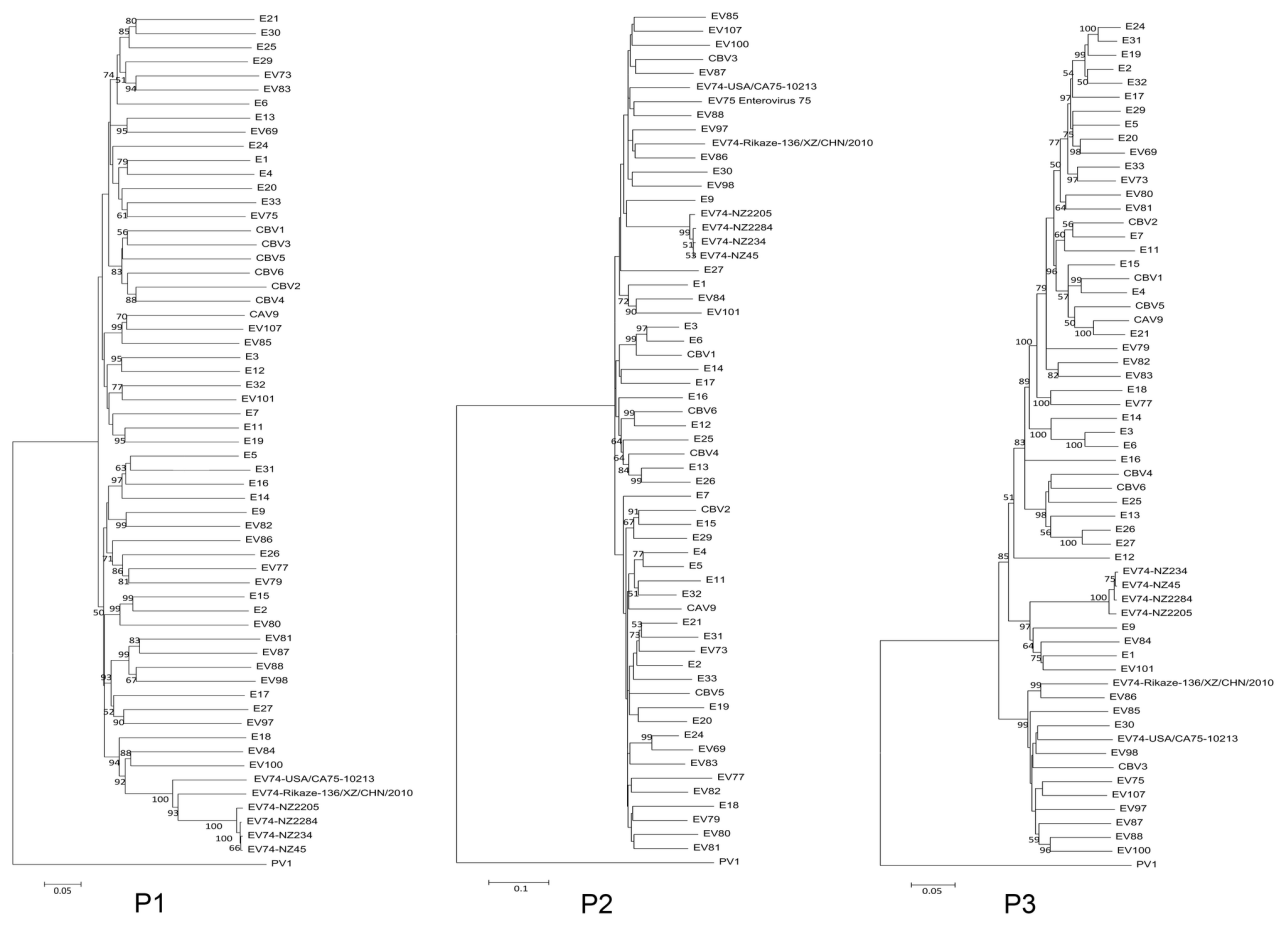
Phylogenetic trees for each region of all available prototype EV B strains. Trees were rooted with PV1. Bootstrap values below 50 were removed. Bars indicate nucleotide substitution distance.

**Figure 3:**
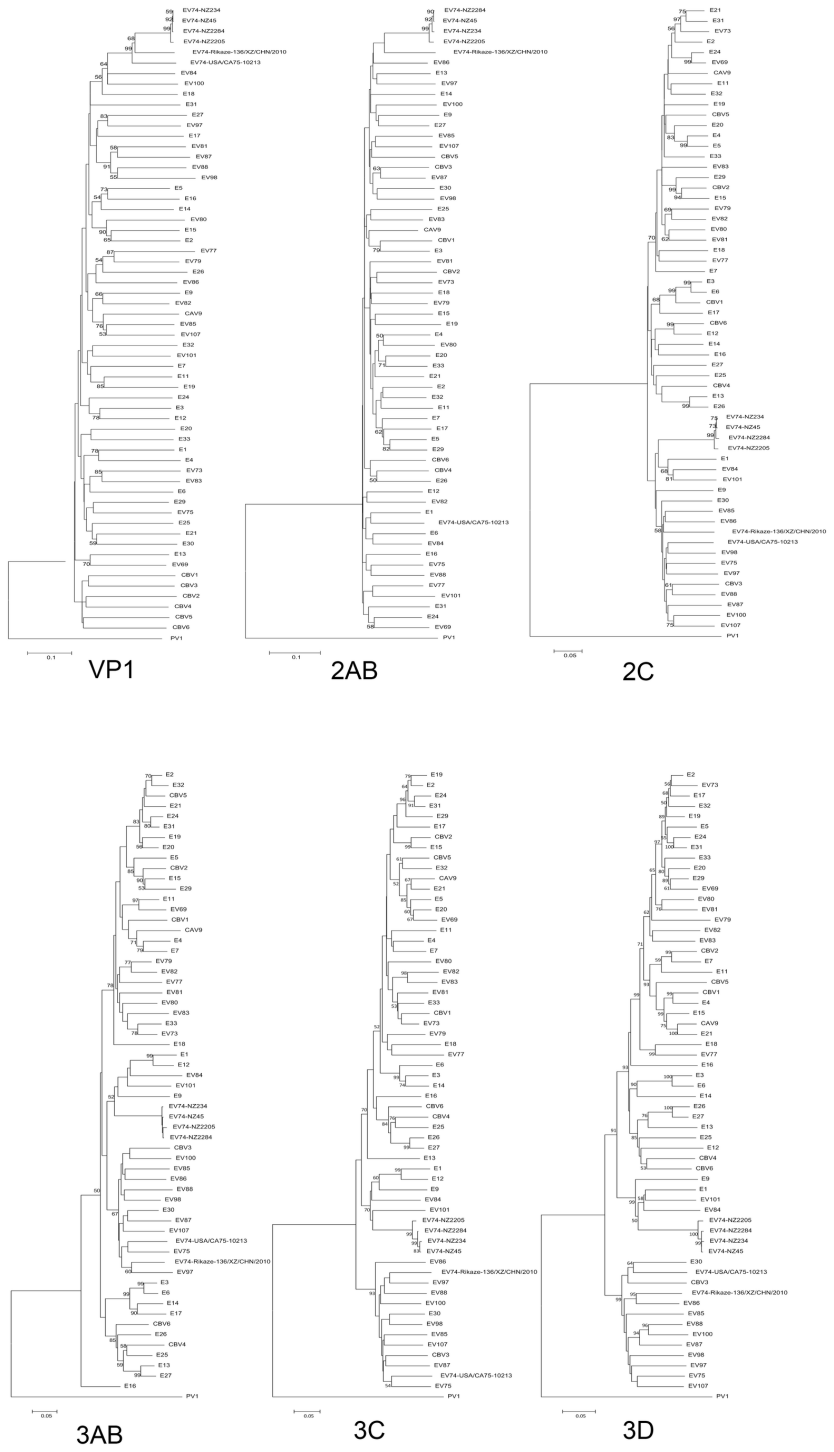
Phylogenetic trees for each genome fragment of all available prototype EV B strains. Trees were rooted with PV1. Bootstrap values below 50 were removed. Bars indicate nucleotide substitution distance.

**Figure 4:**
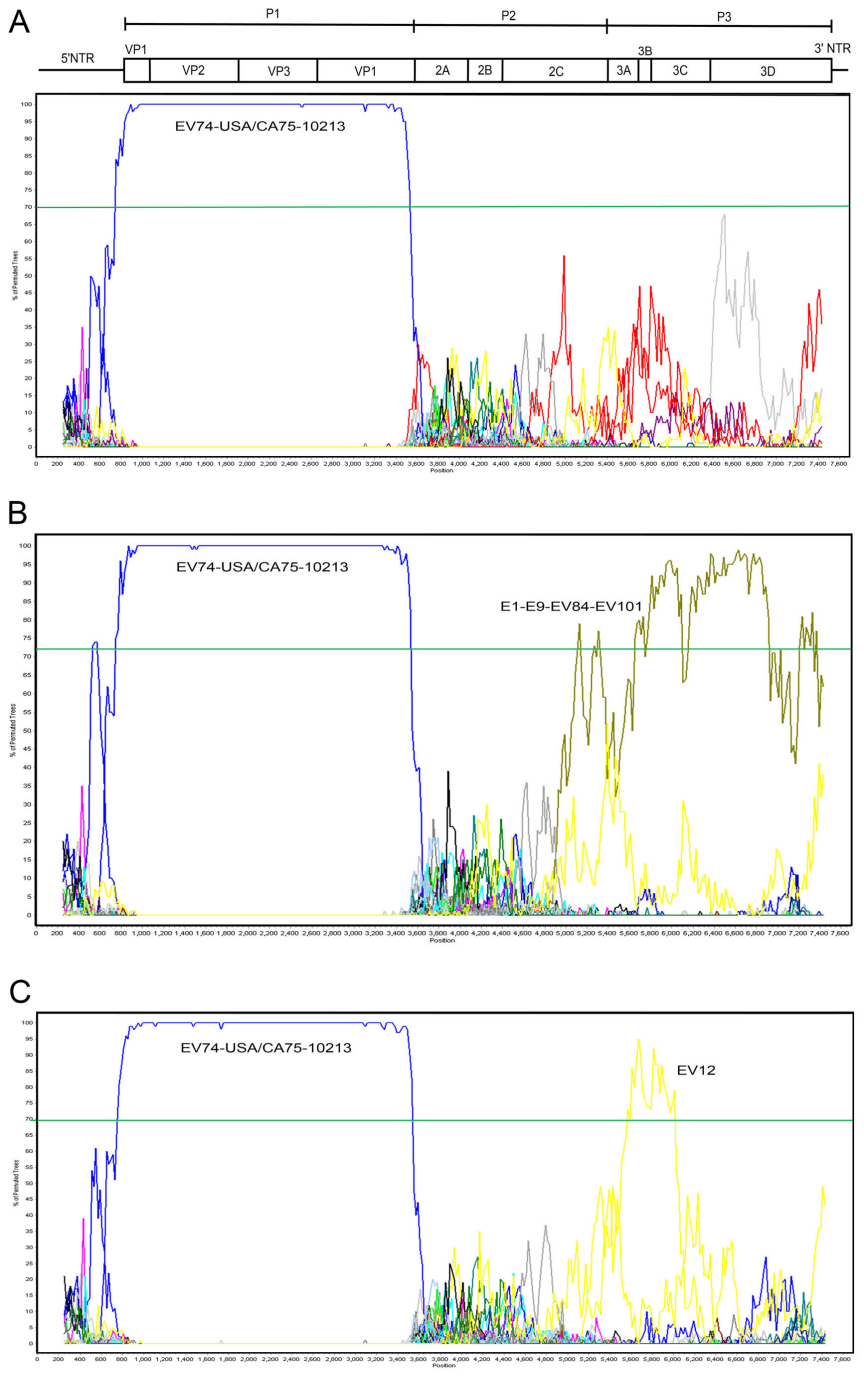
Bootscan analysis of NZ2284 and EV B strains. A, comparison of NZ2284 with all available prototype EV B strains. B, comparison of NZ2284 with all available prototype EV B strains excluding strains E1, E9, EV84 and EV101 but with a consensus sequence of these 4 strains. C, comparison of NZ2284 with all available prototype EV B strains including E12 but excluding strains E1, E9, EV84 and EV101. Schematic above the graph indicates the position of each gene within the EV genome.

Phylogenetic trees were constructed for each gene and the P1, P2 and P3 coding regions (Figure 2) with all available EV B reference genomes (Table 1). All four New Zealand EV74 isolates group closely with both the EV74 reference strains in the P1 region, but do not show a similar grouping in either the P2 or P3 region suggesting a recombination event. No distinguishing relationship to another EV reference strain is observed within the P2 region (bootstrap values < 50) (Figure 3). Examination of the P3 region by both phylogenetics and bootscan analysis (Figure 4) shows the four New Zealand EV74 isolates grouped most closely with E1, E84, E9 and EV101 reference strains. When the 3AB and 3C genes are considered the same phylogenetic relationships are revealed along with E12, although bootstrap values for 3AB relationship is low (Figure 3).

As a contemporary strain is likely to be a closer ancestor, rather than a reference strain, a BLAST search was performed for each gene region to identify the most similar sequences (Table 3). Unsurprisingly the P1 region showed the closest similarity to EV74 strains with the top hit ranging in similarity from 83.4% to 86.5%. Within the P2 and P3 regions greater similarity was observed to contemporary strains although only gene regions 2B and 3B showed similarity higher than 85%. 

**Table 3:** Three closest BLAST hits for each gene region of NZ2284.

**Region**	**Genotype**	**Description**	**Accession Number**	**% Identical Sites**
VP4	EV74	Enterovirus 74 strain IRQ00-10218	AY556061	86.50%
	EV74	Enterovirus 74 strain BAN00-10217	AY556059	85.50%
	EV74	Enterovirus 74 strain CHN97-10215	AY556060	85.00%
VP2	EV74	Human enterovirus 74 strain Rikaze-136/XZ/CHN/2010	JQ397329	84.60%
	EV74	Enterovirus 74 strain BAN00-10217	AY556059	84.20%
	EV74	Enterovirus 74 strain CHN97-10215	AY556060	84.10%
VP3	EV74	Enterovirus 74 strain IRQ00-10218	AY556061	83.70%
	EV74	Enterovirus 74 strain CHN97-10215	AY556060	83.40%
	EV74	Enterovirus 74 strain BAN00-10217	AY556059	83.40%
VP1	EV74	Human enterovirus 74 isolate N-434A	JN204052	84.80%
	EV74	Human enterovirus 74 isolate N-433	JN204051	84.80%
	EV74	Human enterovirus 74 isolate KOR06-332	EF617336	84.10%
2A	CB5	Human coxsackievirus B5 isolate CVB5/SD/09	JX276378	82.90%
	E9	Echovirus E9 strain MSH/KM812/2010	JN596587	82.90%
	CB4	Human coxsackievirus B4 isolate J4	AY831488	82.80%
2B	CB4	Human coxsackievirus B4 isolate F1	AY831498	90.60%
	CB4	Human coxsackievirus B4 isolate J12	AY831496	90.60%
	CB4	Human coxsackievirus B4 isolate F3	AY831499	90.60%
2C	CB3	Human coxsackievirus B3	JN979570	83.00%
	E16*	Human echovirus AMS721	EF155422	82.90%
	E30	Human echovirus 30, isolate CF1495-00	AM237320	82.80%
3A	E6	Human echovirus 6 isolate CF1007_FRA00	FN691457	84.60%
	E30	Human echovirus 30 isolate CF298-81	AM237328	84.60%
	E6	Human echovirus 6 isolate CF621_FRA01	FN691459	84.60%
3B	CA9*	Human enterovirus B strain Cuba35of93	AY466030	95.50%
	CA9*	Human enterovirus B strain Cuba689of93	AY466032	95.50%
	CA9*	Human enterovirus B strain Cuba23of00	AY466033	95.50%
3C	CA9*	Human enterovirus B strain Cuba267of90	AY466029	84.70%
	EV101	Human enterovirus 101 strain CIV03-10361	AY843308	84.30%
	E9	Human echovirus 9 strain Barty	AF524866	83.90%
3D	E1	Echovirus 1 (strain Farouk / ATCC VR-1038)	AF029859	84.60%
	CA9*	Human enterovirus B strain Cuba267of90	AY466029	84.50%
	CA9*	Human enterovirus B strain Cuba689of93	AY466032	84.00%

* Possible genotype determined by BLAST analysis of the VP4-VP1 nucleotide region.

## Discussion

Four New Zealand EV74 viruses were isolated and sequenced showing 98.7% nucleotide sequence identity. These viruses were isolated during a six month interval within 400km of each other. These genetic and epidemiological indicators suggest a common circulating strain. There was no apparent genetic difference between the NZ2284 strain isolated from a patient with AFP, and other isolates from patients with milder symptoms. This is consistent with previous observations that the severity of symptoms does not necessarily correlate with genomic changes in the viral genome [22].

Full genome sequencing of all four isolates revealed recombination had taken place within the non-structural coding region when compared to either of the two prototypic reference strains. This can be expected, as the California strain, USA/CA75-10213, was isolated in 1975, given the time that has elapsed and frequency of recombination among the EV B species [12]. Work by Simmonds et al. suggests the likelihood of recombination occurring somewhere between the VP1 and 3D regions after only one year is almost 40%, while strains isolated more than 10 years apart are almost certain to have undergone a recombination event [23].

During 2010, an EV74 virus was isolated in China, strain Rikaze-136/XZ/CHN/2010 [14]. Despite being isolated during the same year this strain was significantly different (80.9% sequence identity) suggesting a different lineage from the New Zealand isolated strains.

The determination of the sites of recombination in the NZ EV74 isolates is complexand not the focus of this paper. No recombination was observed within the structural region of EV74 (bootstrap value of 100) consistent with previous findings that recombination within this region occurs only rarely [23]. Simplot analysis demonstrates a clear distinction in homology between all EV74 isolates and other EV B strains in the structural region. Distinction of EV serotypes in the non-structural regions is not as clear, especially in the P2 region where it is likely that multiple recombination events have taken place creating a mosaic within this region. Phylogenetic analysis of the P2 region of all EV B prototype strains shows no clear relationship. This is consistent with work mapping phylogeny violations in tree ordering, demonstrating the P2 region is a hotspot for recombination [23].

There is greater sequence homology in the P3 region of the NZ EV74 strains, with a common sequence related to E1, E9, EV84 and EV101 viruses. Again, this situation is complex as when compared to contemporary strains further recombination is evident.

The occurrence and clustering of rare enteroviruses in the human population, such as the four EV74 isolates we reported here, reiterates that on-going surveillance is necessary to determine the epidemiology of these viruses. Furthermore, detailed characterisation of the viral genomes is required to understand the evolution of rare enteroviruses and to assist in the assessment of their potential threat as an emergent disease. The genetic data we report in this study support the notion that there was a cluster of EV74 cases in New Zealand, but do not support the hypothesis that AFP could be caused by a distinct genetic lineage of EV74.
